# Recent advances in the treatment of non-small cell lung cancer with MET inhibitors

**DOI:** 10.3389/fchem.2024.1501844

**Published:** 2024-12-10

**Authors:** Dongna Zhang, Wenying Zhang, He Liu, Pan Liu, Chunxin Li, Yangyang Liu, Jicheng Han, Guangze Zhu

**Affiliations:** ^1^ Department of Clinical Laboratory Medicine, The Affiliated Hospital to Changchun University of Chinese Medicine, Changchun, China; ^2^ Key Laboratory of Jilin Province for Traditional Chinese Medicine Prevention and Treatment of Infectious Diseases, College of integrative medicine, Changchun University of Chinese Medicine, Changchun, China

**Keywords:** NSCLC, MET inhibitors, drug discovery, structure-activity relationships, targeted therapy

## Abstract

Recently, research into the oncogenic driver genes associated with non-small cell lung cancer (NSCLC) has advanced significantly, leading to the development and clinical application of an increasing number of approved therapeutic agents. Among these, small molecule inhibitors that target mesenchymal-epithelial transition (MET) have demonstrated successful application in clinical settings. Currently, three categories of small molecule MET inhibitors, characterized by distinct binding patterns to the MET kinase region, have been developed: types Ia/Ib, II, and III. This review thoroughly examines MET’s structure and its crucial role in NSCLC initiation and progression, explores discovery strategies for MET inhibitors, and discusses advancements in understanding resistance mechanisms. These insights are anticipated to enhance the development of a new generation of MET inhibitors characterized by high efficiency, selectivity, and low toxicity, thereby offering additional therapeutic alternatives for patients diagnosed with NSCLC.

## 1 Introduction

**FIGURE 1:**
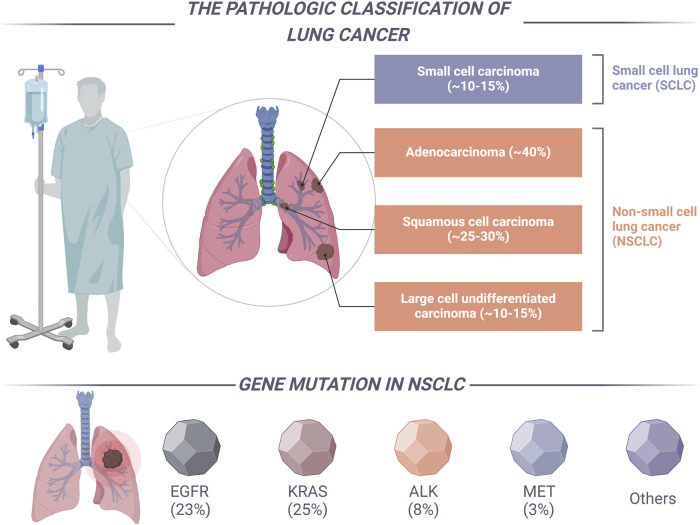
The categorization of lung cancer and the primary driver mutation types associated with NSCLC.

According to data from the International Agency for Research on Cancer (IARC), the global incidence of newly diagnosed cancer cases reached 19.6 million in 2022. Concurrently, cancer-related mortality accounted for approximately 9.7 million deaths worldwide. These statistics underscore the emergence of cancer as a critical public health issue on a global scale (Bray et al., 2024). In 2022, lung cancer accounted for over 12% of all new cancer diagnoses, with an estimated 2.48 million new cases reported, and it was associated with approximately 1.81 million deaths globally (Abu Rous et al., 2023). As shown in Figure 1, Lung cancer is a diverse group of tumors mainly categorized into two types based on cell origin: non-small cell lung cancer (NSCLC) and small cell lung cancer (SCLC). Notably, NSCLC represents the predominant variant, comprising approximately 85%–90% of all lung cancer cases. Due to the often insidious nature of the early clinical manifestations of NSCLC, clinical data suggests that approximately 75% of patients are diagnosed at an advanced stage of the disease. In patients with advanced NSCLC, surgical intervention is considered the primary treatment modality. However, approximately 40% of NSCLC patients experience recurrence and metastasis following complete tumor resection, which contributes to a diminished 5-year survival rate (Imyanitov et al., 2021). Given the restricted applicability of surgical intervention for patients with advanced NSCLC, clinical practice frequently resorts to chemotherapy and radiation therapy utilizing platinum-based agents as the primary treatment approach for this patient population. Nevertheless, the prognosis for NSCLC patients remains suboptimal due to constraints on radiation dosage, the adverse side effects associated with chemotherapy, and the emergence of drug resistance (Wang L. et al., 2023; Wang N. et al., 2023). Consequently, it is imperative to identify and investigate treatment strategies that exhibit enhanced selectivity and a diminished occurrence of adverse reactions. The advent of genetic testing technology is particularly promising, as it facilitates the identification of specific targets and the development of targeted therapies, thereby heralding a new era in the management of NSCLC.

Targeted therapy for NSCLC serves as a prominent illustration of precision medicine, wherein distinct subtypes characterized by specific driver mutations inform the development of targeted treatment strategies (Salgia et al., 2021). The National Comprehensive Cancer Network (NCCN) guidelines delineate specific clinically relevant genes, including the epidermal growth factor receptor (EGFR), anaplastic lymphoma kinase (ALK), c-ros oncogene 1 tyrosine kinase (ROS1), v-Raf murine sarcoma viral oncogene homolog B1 (BRAF), human epidermal growth factor receptor 2 (HER2), and mesenchymal-epithelial transition factor (MET), along with other established and frequently implicated driver genes (Figure 1) (Alexander et al., 2020). In the past decade, significant advancements have been made in the research of driver genes associated with NSCLC. The clinical application of a range of targeted therapies directed at key driver genes has resulted in improved survival outcomes for patients with NSCLC. The MET gene serves as a critical oncogenic driver in NSCLC and is recognized as a significant therapeutic target, following EGFR, ALK, and ROS1 (Duma et al., 2019). The advancement of pharmacological agents aimed at MET gene mutations has attracted considerable interest among medicinal chemists. In recent years, the FDA has granted approval for small molecule inhibitors, including capmatinib, savolitinib, and tepotinib, for the treatment of patients with metastatic NSCLC who possess MET exon 14 skipping mutations (Wu and Lin, 2022).

This review offers a thorough overview of the structure, biological functions, and dysregulation of the MET receptor. The focus is particularly on recent advancements in the development of MET inhibitors, in addition to elucidating the mechanisms underlying resistance to these inhibitors. Furthermore, the review discusses clinical strategies for combination therapies involving MET inhibitors and explores innovative treatment modalities aimed at targeting MET, including the use of PROTAC technology. A comprehensive understanding of the advancements in MET inhibitors for the treatment of NSCLC may enhance the discovery and development of novel, more effective, and less toxic inhibitors.

## 2 HGF/MET signaling pathway

**FIGURE 2:**
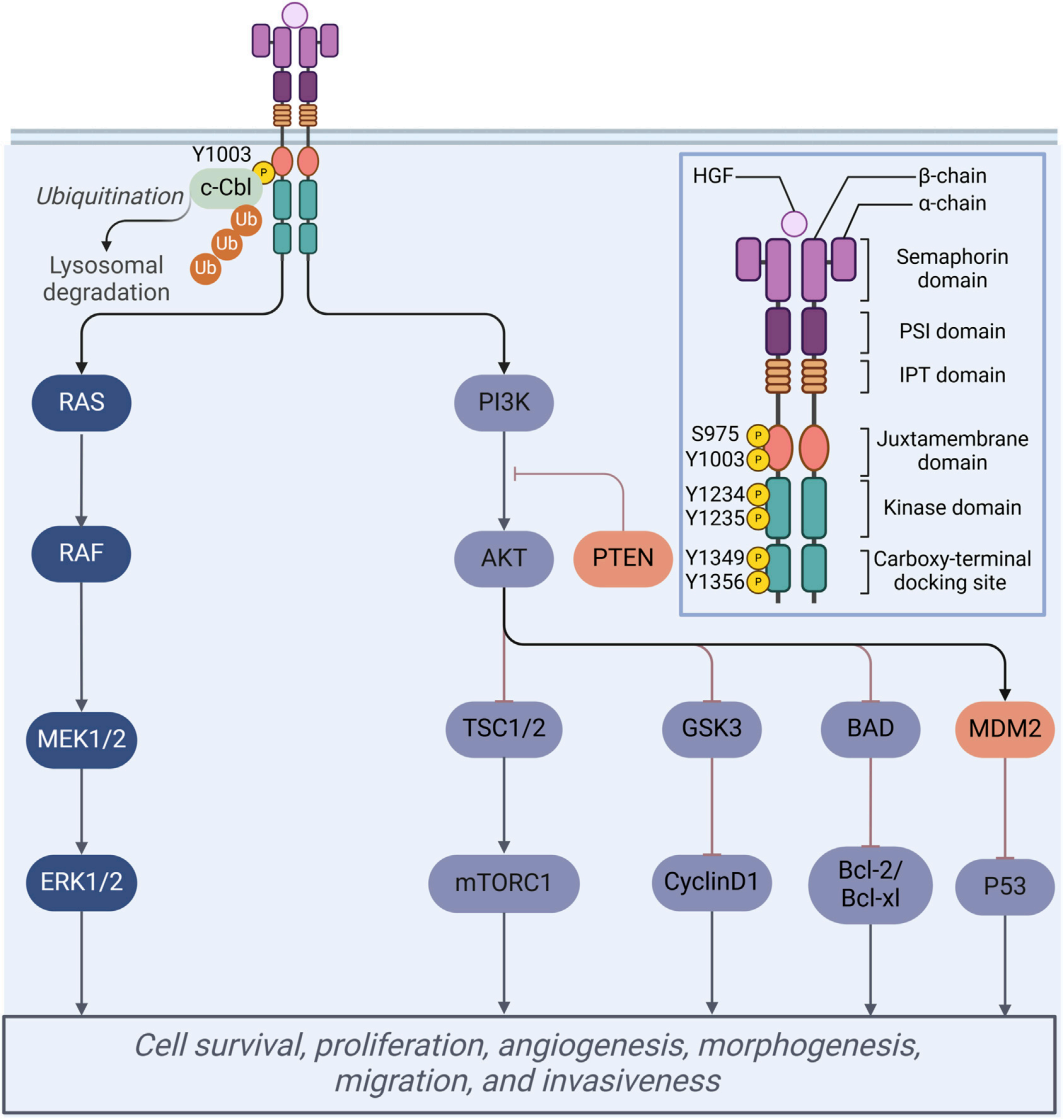
Schematic representation of the MET protein domain structure. Mechanisms of multiple cellular processes regulated by HGF/Met signaling.

The MET proto-oncogene, located on the long arm of chromosome 7 in humans (7q21-31), encodes a protein that extends over 120 kilobases and consists of 21 exons along with 20 introns. This protein functions as a transmembrane receptor and is classified within the receptor tyrosine kinase (RTK) family (Koch et al., 2020). Structurally, the MET receptor is composed of several distinct domains, including an extracellular domain, a transmembrane domain, and an intracellular region that encompasses a juxtamembrane domain, a catalytic kinase domain, and a carboxy-terminal tail (Figure 2). The extracellular domain of the MET receptor comprises three distinct structural domains: the N-terminal fragment, which adopts a conformation resembling a large signaling proten-like (SEMA) domain that encompasses the entirety of the alpha subunit and a portion of the beta subunit; the plexin-semaphorin-integrin (PSI) domain, situated downstream of the SEMA domain and characterized by the presence of four disulfide bonds; and the PSI domain, which is linked to four immunoglobulin-plexin-transcription factor (IPT) domains (Pothula et al., 2020).

Under physiological conditions, mature hepatocyte growth factor (HGF) demonstrates a high-affinity interaction with the MET receptor, specifically within the extracellular ligand-binding domain (Yin et al., 2019). This interaction initiates the phosphorylation of tyrosine residues Tyr1234 and Tyr1235 located in the tyrosine kinase domain. The phosphorylation of these residues induces a conformational change in the receptor, which subsequently facilitates the phosphorylation of two additional docking tyrosine residues, Tyr1349 and Tyr1356, at the carboxy terminus (Mulcahy et al., 2020). The activation of these residues enables the recruitment of various downstream signaling molecules, thereby contributing to a homologous signaling cascade. Upon the activation of the HGF/MET signaling pathway, the downstream signaling cascades, specifically the PI3K/AKT/mTOR and RAS/RAF pathways, are subsequently activated, thereby facilitating a range of physiological functions (Figure 2). These pathways are essential to a range of cellular processes, encompassing proliferation, migration, inhibition of apoptosis, and angiogenesis, and function within both cytoplasmic and nuclear compartments (Cheng and Guo, 2019).

## 3 MET dysregulation in NSCLC

**FIGURE 3:**
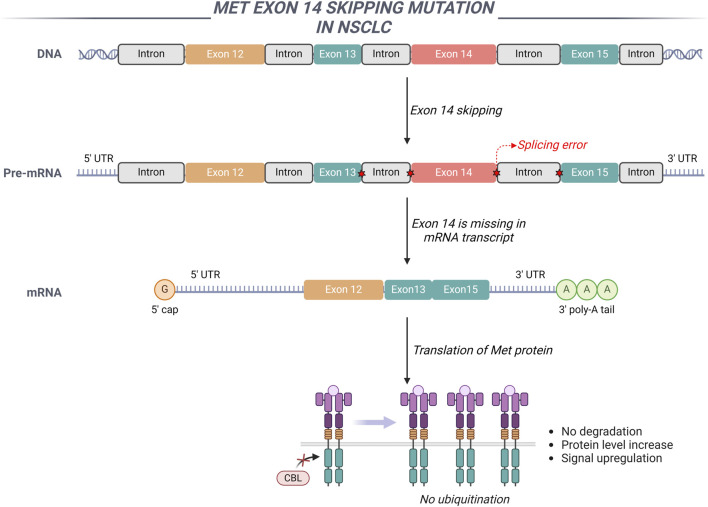
The involvement of MET exon 14 in NSCLC is illustrated through a schematic representation of the genomic regions adjacent to MET exon 14. The exclusion of MET exon 14 results in the upregulation of MET signaling pathways.

The dysregulation of the HGF/MET signaling pathway, attributable to exon 14 skipping mutations, amplifications, overexpression, and fusion events, plays a significant role in the initiation and progression of various malignant tumors (Remon et al., 2023). The extracellular domain encoded by exon 14 of the MET gene serves as a crucial negative regulatory region, playing a significant role in the ubiquitination and subsequent degradation of the MET protein (Guo et al., 2020). The intronic region adjacent to exon 14, along with exon 14 itself, or a complete genomic deletion of exon 14, disrupts the splicing mechanism of MET gene transcription. This deletion inhibits the processes of ubiquitination and endocytosis, consequently impairing the degradation of the MET receptor protein (Drusbosky et al., 2021). Such impairment leads to excessive activation of MET-mediated signaling pathways, thereby promoting cellular proliferation and tumor growth (Figure 3). In NSCLC, the overall incidence rate of MET exon 14 skipping mutations varies between 2.5% and 6.0%. These mutations do not co-occur with other driver genes such as EGFR and ALK, suggesting that they function as independent oncogenic drivers. Nonetheless, MET exon 14 skipping mutations can coexist with MET gene amplification and protein overexpression (Huang et al., 2020).

MET gene amplification is characterized by an increase in the copy number of the MET gene, encompassing both whole chromosome duplication and localized gene duplication (Tyler et al., 2022). Specifically, whole chromosome duplication refers to the presence of multiple copies of chromosome 7 within tumor cells. The amplification of the MET gene frequently occurs in conjunction with mutations in other oncogenes, including EGFR and KRAS (Yang et al., 2024). Current research indicates that MET amplification may not serve as a primary driver gene in NSCLC. Furthermore, a significant correlation exists between MET amplification and the activation of other driver genes, such as EGFR and KRAS, implying that MET amplification may represent one of the mechanisms contributing to acquired resistance in NSCLC characterized by EGFR mutations (Wang et al., 2019). Research has demonstrated that 15%–20% of patients with acquired resistance to EGFR inhibitors exhibit detectable amplification of the MET gene. Moreover, MET gene amplification is frequently associated with a poorer prognosis in patients with NSCLC (Shi et al., 2016).

Abnormal overexpression of either MET or HGF may result in prolonged activation of these pathways, potentially contributing to the initiation and progression of tumors (Chen et al., 2022). Research has demonstrated that the overexpression of HGF and its receptor, Met, in NSCLC is significantly correlated with the formation of lymphatic vessels (Kajiya et al., 2005). In pulmonary adenocarcinoma, the prevalence of MET overexpression can reach up to 65%; however, only 10% of cases exhibiting MET overexpression also present with mutations in the MET gene (Wang N. et al., 2024). Consequently, it is plausible that MET overexpression may not serve as the primary oncogenic driver, but rather as a secondary event arising from the activation of alternative driver genes, thereby facilitating tumor growth (Wang N. et al., 2023).

The advancement of genomics and proteomics has facilitated a transition in the treatment of NSCLC towards a personalized approach informed by the identification of driver genes (Bi et al., 2023). Over the past decade, numerous therapeutic strategies aimed at modulating aberrant HGF/MET signaling pathways have been documented, primarily encompassing three approaches: i) the utilization of biological agents or neutralizing antibodies to obstruct the extracellular interaction between MET and HGF. Especially, HGF antagonists, like liromalimumab and pemigatinib, competitively bind to the MET receptor, blocking its interaction with HGF without activating the receptor (Kim and Kim, 2017). The anti-MET monoclonal antibody Onartuzumab possesses the ability to inhibit the formation of the MET dimer, thereby attenuating downstream signaling and intracellular signal transduction, which ultimately contributes to the inhibition of tumor growth (Kim et al., 2021); ii) Small molecule inhibitors targeting MET kinase bind to the catalytic core region of MET, thereby inhibiting the phosphorylation of intracellular tyrosine kinases and disrupting the transmission of downstream signaling pathways (Spagnolo et al., 2023); iii) the inhibition of downstream signaling pathway proteins associated with HGF/MET to disrupt signal transduction (He et al., 2021). In recent decades, significant progress has been made in the development of small molecule inhibitors targeting the MET kinase, leading to the advancement of numerous candidates into clinical trials, several of which have subsequently received market approval (Ye et al., 2021). The following chapters will focus on the advancements in small molecule inhibitors specifically aimed at the tyrosine kinase domain of MET.

## 4 Small-molecule inhibitors of MET

To date, numerous MET inhibitors have been documented for the clinical management of NSCLC (Table 1) (Leonetti et al., 2019). Existing MET inhibitors can be categorized into three distinct classes based on their unique binding modes with biological target. Type I inhibitors interact with the active conformation of the kinase within the ATP binding pocket, further subdivided into non-selective type Ia and selective type Ib inhibitors. Type II inhibitors engage with the inactive conformation of the kinase within the ATP pocket. Finally, Type III inhibitors bind outside the ATP pocket, employing a non-ATP competitive allosteric mechanism (Mazieres et al., 2023). In light of the increasing prevalence of drug resistance, there has been a notable surge in research focused on novel MET inhibitors, complementing the previously discussed clinically available inhibitors (Jørgensen et al., 2024). The subsequent sections of this chapter will offer a comprehensive overview of the advancements in research and the structure-activity relationships associated with representative MET inhibitors from the three categories previously mentioned.

**TABLE 1:** Overview of clinical approved MET inhibitors for the treatment of NSCLC.

Types of MET inhibitors	Drugs	Chemical structures	Phase	Clinical trial number	Status
Ia	Crizotinib (**1**)	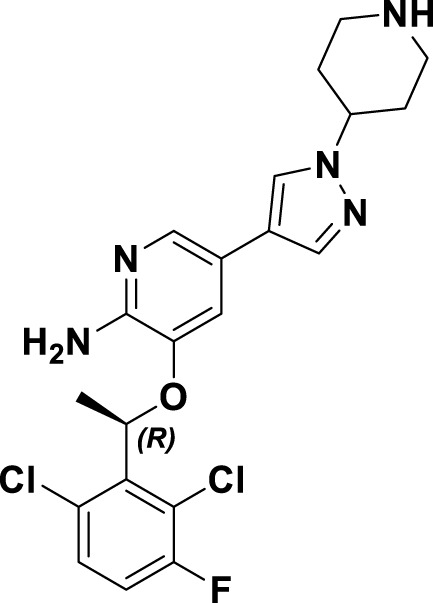	III	NCT02767804 NCT04632758 NCT03052608 NCT06569420 NCT02838420 NCT02075840	Active
			I/II	NCT01712217 NCT01970865	Completed
			IV	NCT03672643	Recruiting
			II	NCT01945021 NCT02183870 NCT03088930 NCT00932451	Completed
			III	NCT06140836 NCT06082635 NCT04603807	Recruiting
			I/II	NCT05384626	Recruiting
			II	NCT01500824	Withdrawn
			IV	NCT05160922	Active
			I	NCT01579994 NCT01998126	Completed
			I/II	NCT02584634	Terminated
			II	NCT04322578	Recruiting
			III	NCT02737501 NCT01639001 NCT00932893 NCT01828112	Completed
Ib	Capmatinib (**2**)	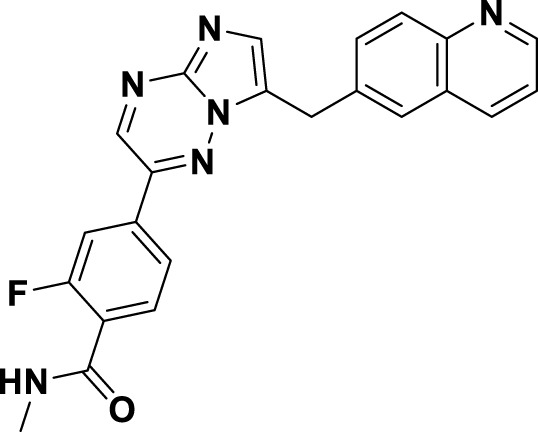	II	NCT04460729 NCT05567055 NCT03240393	Withdrawn
			II	NCT02414139 NCT03647488 NCT02750215 NCT01610336 NCT02276027	Completed
			II	NCT04677595	Active
			IV	NCT05110196	Recruiting
			I/II	NCT05488314	Active
			III	NCT04427072	Completed
			III	NCT04816214	Terminated
			I	NCT01911507 NCT01324479	Completed
			II	NCT05642572 NCT03040973	Recruiting
			I	NCT02468661	Terminated
	Savolitinib (**3**)	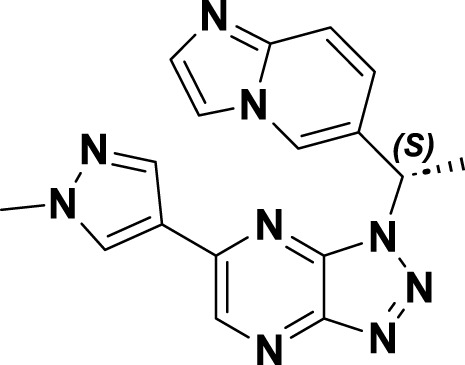	III	NCT04923945	Recruiting
			II	NCT02117167	Completed
			II	NCT04322890	Recruiting
			II	NCT03778229 NCT05163249 NCT03833440 NCT04606771 NCT03944772	Active
			III	NCT05261399	Recruiting
			II	NCT04322578	Recruiting
			I	NCT02374645	Completed
			I	NCT02143466	Active
	SAR125844 (**4**)	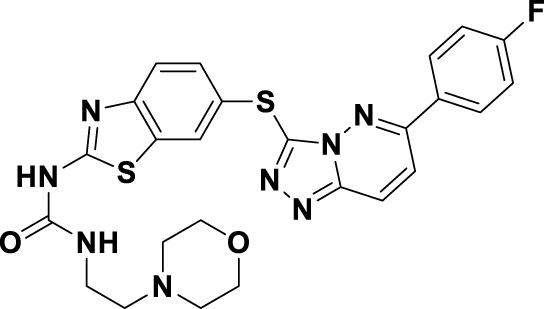	I	NCT01391533	Completed
			I	NCT01657214	Completed
			II	NCT02435121	Completed
	Bozitinib (**5**)	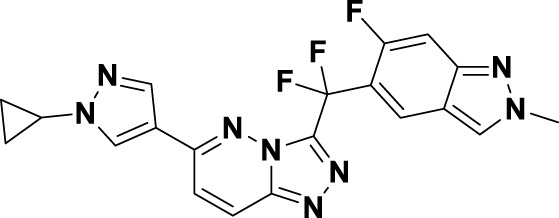	II	NCT03175224	Recruiting
			I/II	NCT04743505 NCT06343064	Recruiting
			I	NCT02896231	Completed
			I/II	NCT03655613	Terminated
	Tepotinib (**6**)	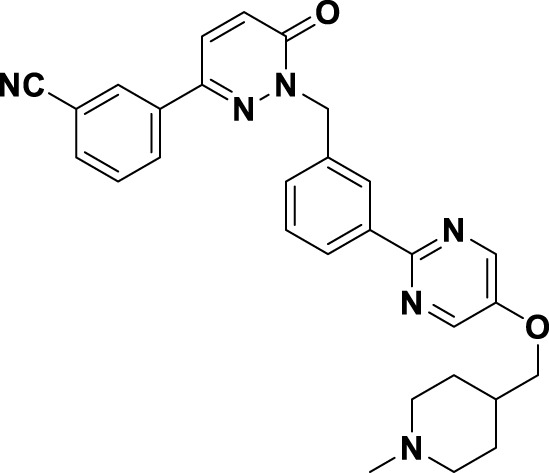	I/II	NCT04739358	Terminated
			I/II	NCT06083857	Recruiting
			II	NCT02864992 NCT03940703	Active
			I/II	NCT01982955	Completed
	ABN401 (**7**)	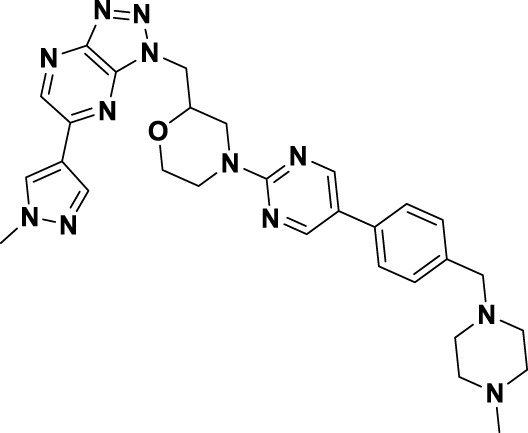	II	NCT05541822	Recruiting
			I	NCT04052971	Recruiting
			I	NCT05248074	Completed
	Elzovantinib (**8**)	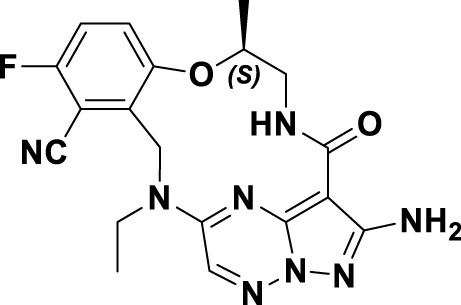	I/II	NCT03993873	Active
	Glumetinib (**9**)	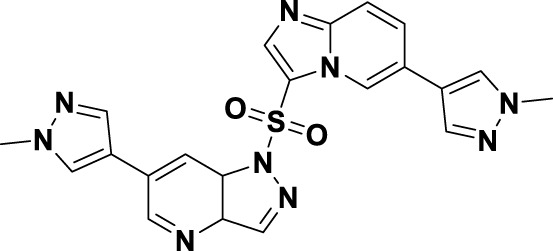	I/II	NCT04270591	Unknown
II	Cabozantinib (**10**)	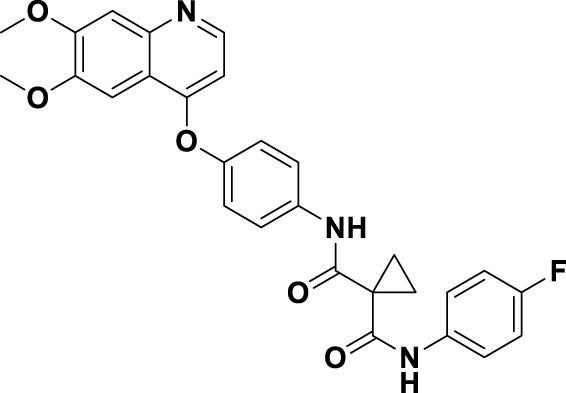	II	NCT02132598	Terminated
			I/II	NCT00596648	Completed
			I	NCT04173338	Terminated
			II	NCT01639508 NCT05613413	Recruiting
			II	NCT00940225 NCT05200143 NCT03468985 NCT01866410 NCT02795156	Completed
			III	NCT04471428	Active
			I/II	NCT04151563	Withdrawn
			II	NCT04310007 NCT01708954	Active
	Foretinib (**11**)	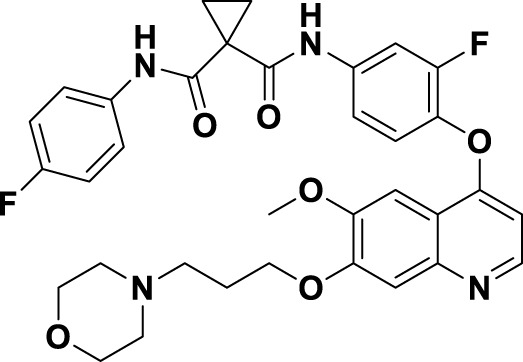	II	NCT02034097	Withdrawn
			I/II	NCT01068587	Completed
	Glesatinib (**12**)	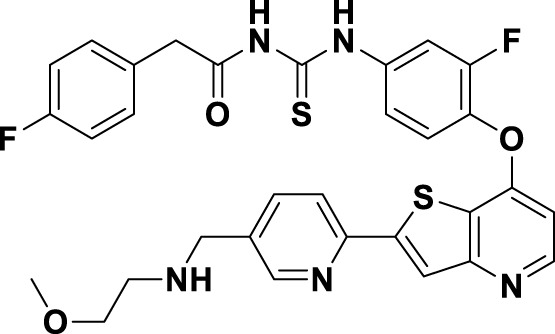	II	NCT02954991	Terminated
	Merestinib (**13**)	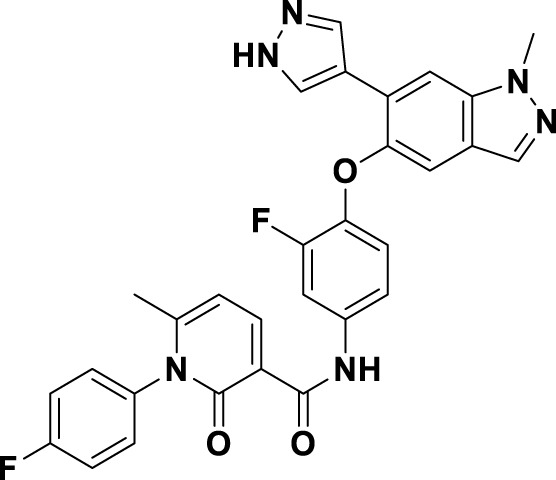	II	NCT02920996	Terminated
	Ningetinib (**14**)	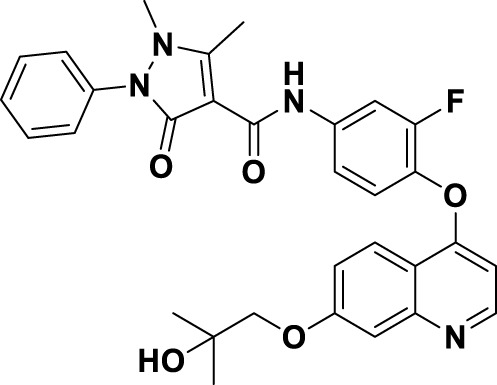	II	NCT03838848	Terminated
	BMS-777607 (**15**)	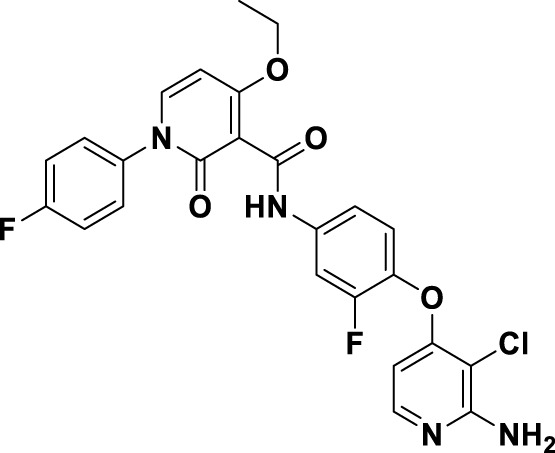	I/II	NCT00605618	Completed
			I	NCT01721148	Completed
III	Tivantinib (**16**)	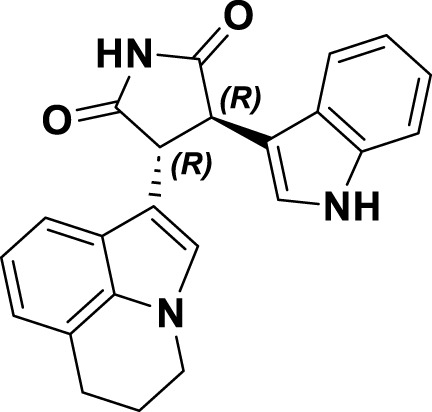	III	NCT01377376	Terminated

### 4.1 Type Ia inhibitors of MET

Type I small molecule inhibitors that target MET engage the active conformation of the protein’s ATP-binding pocket in a ‘U-shaped’ orientation, commonly referred to as ‘DFG-in’ (Sun et al., 2019). Type I inhibitors are further categorized into subclasses Ia and Ib, depending on their interaction with the G1163 residue, which is solvent-exposed. Notably, Type Ia inhibitors interact hydrophobically with the G1163 residue, whereas type Ib inhibitors do not (Zhang et al., 2019).

**FIGURE 4:**
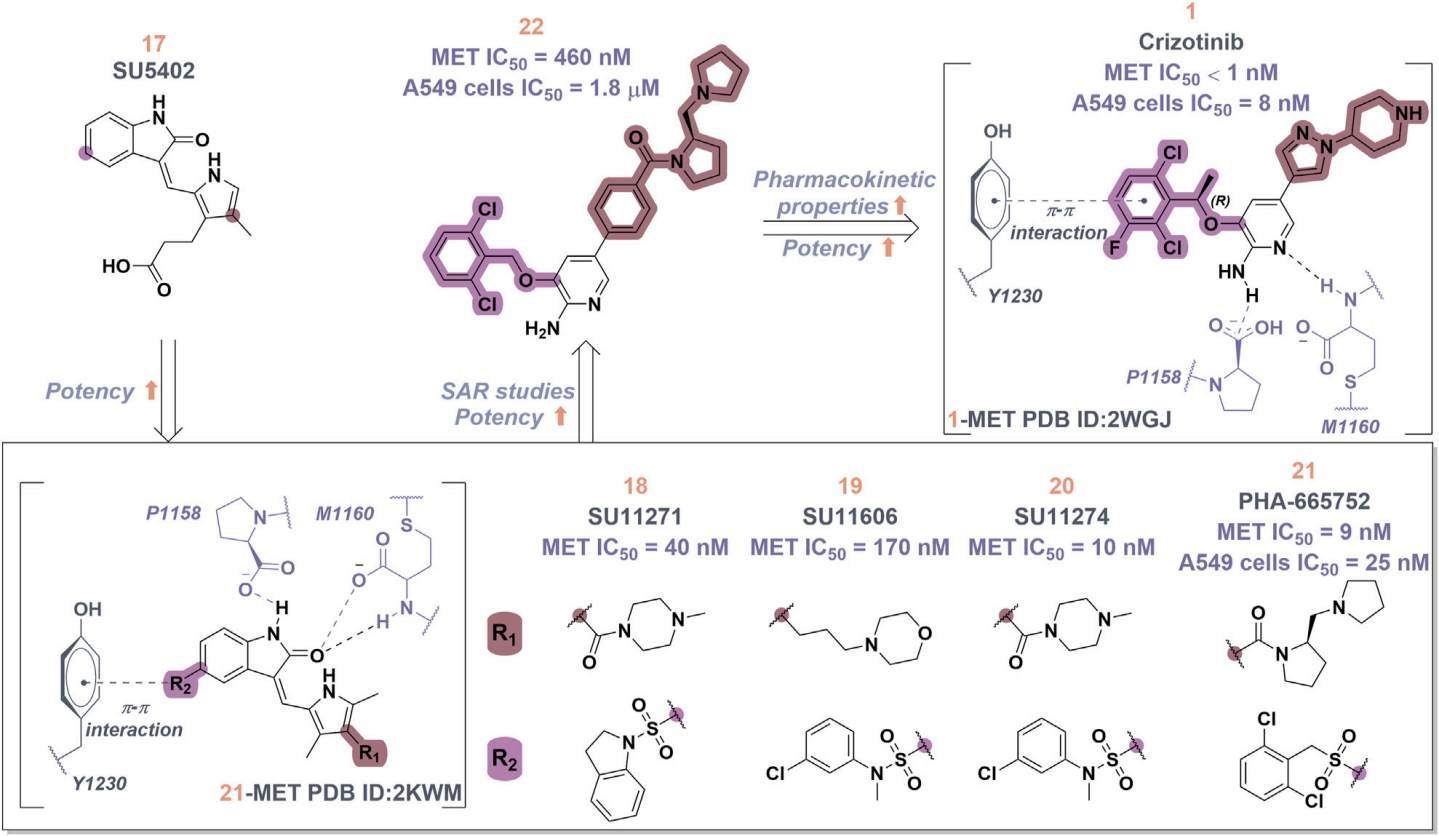
The identification and SAR studies of the pyrrole indolinone derivatives **17–21**, 2-aminopyridine derivative **22**, and crizotinib (**1**). Mauve dashed lines represent hydrogen bonds with key amino acids, and faint gray dashed lines indicate π-π interactions.

Crizotinib (**1**), a prototypical type Ia inhibitor of MET, was identified from the precursor molecule SU5402 (**17**) via extensive structural modifications (Cui et al., 2011). Figure 4 demonstrate that strategically adding groups at the C5 position of the indolone and on the pyrrole ring of **17** can greatly improve the molecule’s inhibitory activity against the MET kinase, leading to the development of compounds SU11271 (**18**), SU11606 (**19**), and SU11274 (**20**). The substitution of the *N*-methylsulfonamide in compound **20** with a more flexible methylenesulfonyl group led to the synthesis of PHA-665752 (21), which demonstrated low nanomolar cellular antiproliferative and inhibitory activities against the MET kinase (Michaelides et al., 2023). The co-crystallization of **21** with the MET kinase (PDB ID: 2KWM) revealed that the benzylsulfonate moiety underwent self-folding to engage in interactions with the Y1230 residue, rather than directly associating with the hydrophobic region of the MET kinase. However, the poor pharmacokinetics of **21** hindered its clinical development, requiring structural optimization. Based on the binding mode analysis of **21** with MET, derivatives were identified by substituting its core with pyrido [2,3-*b*]indole or aminopyridine. Compound **22**, featuring a 2-aminopyridine core, more effectively occupies the adenine pocket of the MET kinase and establishes a hydrogen bond network with residue M1160 compared to **21**. Researchers found that the 2,6-dichlorophenoxy substituent significantly improved π-π interactions with the Y1230 residue in the A ring of the MET structure through detailed structure-activity relationship studies. Concurrently, the incorporation of a hydrophilic group at the tail region of the molecule further improved both the potency and pharmacokinetic properties, ultimately leading to the development of **1**. Co-crystal analysis of compound **1** with MET kinase (PDB ID: 2WGJ) shows that the 2-aminopyridine forms hydrogen bonds with residues P1158 and M1160, while the 2,6-dichloro-3-fluorophenyl group interacts with Y1230 through a π-π interaction. The α-methyl group in the linkage between the 2,6-dichloro-3-fluorophenyl moiety and the parent nucleus facilitates interactions within the hydrophobic cavity constituted by residues V1092, L1157, K1110, and A1108. Additionally, it enhances the rigidity of the substituted phenyl group. This increased rigidity facilitates the establishment of additional electrostatic interactions between the substituted phenyl group and D1222. Furthermore, **1** shows a conserved hydrophobic interaction with M1211, potentially explaining its limited kinase selectivity.

To date, numerous medicinal chemists have conducted comprehensive investigations into the structure-activity relationship of **1** (Yadav et al., 2024). The specific findings are as follows: i) Substituting 2-aminopyridine with alternative nitrogen-containing aromatic heterocycles, such as quinoline, pyridine, and 3-amino-pyrazine, results in a reduction of biological activity; ii) Substituting the oxygen atom in the linking region with either a sulfur atom or an amide bond leads to a decrease in potency. This reduction may be attributed to the alteration in the linking region, which impedes the formation of interactions between the substituted phenyl group and the methionine residue of the protein, thereby affecting the overall efficacy of the molecule; iii) The judicious substitution of hydrophilic groups can effectively sustain biological activity.

### 4.2 Type Ib inhibitors of MET

In contrast to type Ia inhibitors, type Ib inhibitors exhibit penetration into the solvent-accessible region adjacent to the residues D1222, Y1230, and R1228, thereby establishing a more substantial π-π interaction with Y1230 (Fujino et al., 2020). This interaction is instrumental in enhancing selectivity and increasing the inhibitory activity against the MET kinase. As shown in Table 1 (Rocco et al., 2024), several type Ib inhibitors targeting the MET kinase have been approved for clinical use, including capmatinib (**2**), savolitinib (**3**), SAR125844 (**4**), bozitinib (**5**), tepotinib (**6**), ABN401 (**7**), elzovantinib (**8**), and glumetinib (**9**).

**FIGURE 5:**
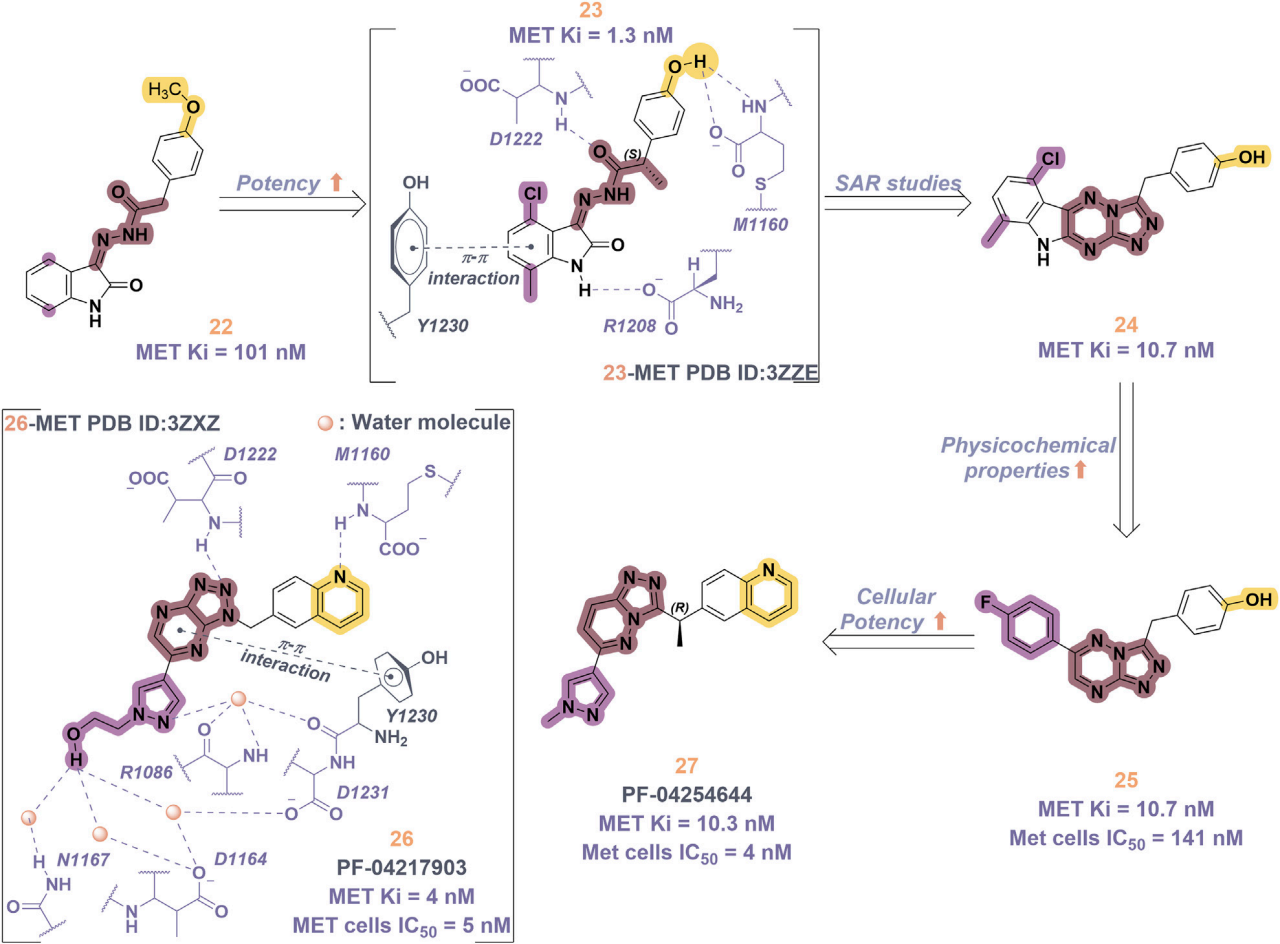
The identification and SAR studies of quinoline-based Type Ib inhibitors 26 and 27 of Met are presented. The essential amino acid residues that mediate interactions between the small molecule and the MET protein are depicted. Mauve dashed lines represent hydrogen bonds with key amino acids, and faint gray dashed lines indicate π-π interactions.

Compound **2**, recognized as the first highly selective MET inhibitor, has been granted approval for the treatment of metastatic NSCLC associated with MET exon 14 skipping mutations (Wolf et al., 2020). *In vitro* studies demonstrate that **2** displays considerable efficacy against MET (IC_50_ = 0.13 nM), as well as against MET-mediated signal transduction and tumor activity at low nanomolar concentrations. Unfortunately, the specific drug discovery pathway leading to the development of **2** has not been publicly disclosed. Recent clinical studies have indicated that the objective response rate (rwORR) of capmatinib as a first-line treatment is 73.4%, while the disease control rate (rwDCR) is 95%. Additionally, the median time to treatment discontinuation (mTTD) is reported to be 19.1 months. Although the median progression-free survival (rwPFS) and median overall survival (mOS) have not yet been reached, the 18-month rates for progression-free survival and overall survival are 68% and 92.6%, respectively. The research has demonstrated that capmatinib, when administered as a first-line treatment for NSCLC with MET exon 14 skipping mutations, yields a superior tumor response, a reduced risk of disease progression, and an extended survival rate compared to regimens based on chemotherapy and immunotherapy (Wu Y. et al., 2021; Dong et al., 2022). As shown in Figure 5, several MET inhibitors utilizing quinoline groups as ligands for binding to the hinge region of the MET kinase have been documented, including PF-04217903 (**26**) (Zou et al., 2012) and PF-04254644 (**27**) (Cui et al., 2013). Initially, compound **22** was recognized as a selective Ib-type MET inhibitor. Subsequently, through the methylation and modification of the core structure of **22**, a more potent MET inhibitor, designated as compound **23**, was identified, exhibiting an IC_50_ value of 1.3 nM. The co-crystal structure of **23** in complex with MET (PDB ID: 3ZZE) demonstrates that the phenolic group within **23** can interact with the M1160 residue located in the hinge region of MET, while the core moiety establishes a robust π-stacking interaction with the critical residue Y1230. Subsequently, compound **24**, which incorporates a tetracyclic aromatic framework, was designed based on the coplanar conformation of **23**. However, the non-pharmacological properties of **24** indicated the need for further structural optimization. Consequently, the tetracyclic framework was deconstructed into various bicyclic frameworks, resulting in the synthesis of compound **25**. Structure-activity relationship studies indicate that the electron-deficient characteristics of aromatic heterocyclic parent nuclei, which contain multiple nitrogen atoms, are significantly correlated with the interaction strength between MET inhibitors and the Y1230 residues. This relationship is closely linked to the efficacy and specificity of small-molecule ligands that interact with the MET receptor. Compound **25** was further optimized through the substitution of the phenol group with a quinoline moiety and the replacement of the p-fluorophenyl group with a pyrazolyl ethanol group, resulting in the synthesis of compounds **26** and **27**, which exhibited enhanced enzyme inhibitory and cellular inhibitory activities. The co-crystal structure of **26** with MET kinase (PDB ID: 3ZXZ) reveals that the quinoline nitrogen forms a hydrogen bond with M1160, while the triazolopyrazinyl moiety participates in a π-π stacking interaction with Y1230. Furthermore, the N atom and -OH group of the pyrazole moiety in **26** are capable of forming a water-mediated polyhydrogen bond network with the MET kinase (Cui et al., 2012). Currently, **26** has progressed to phase I clinical trials for the treatment of advanced solid tumors (Parikh and Ghate, 2018). In contrast, **27**, which shares structural similarities with **26**, has demonstrated cardiac-related toxicity attributed to its insufficient selectivity for the phosphodiesterase family, thereby hindering its advancement in clinical development (Knauf et al., 2018).

**FIGURE 6:**
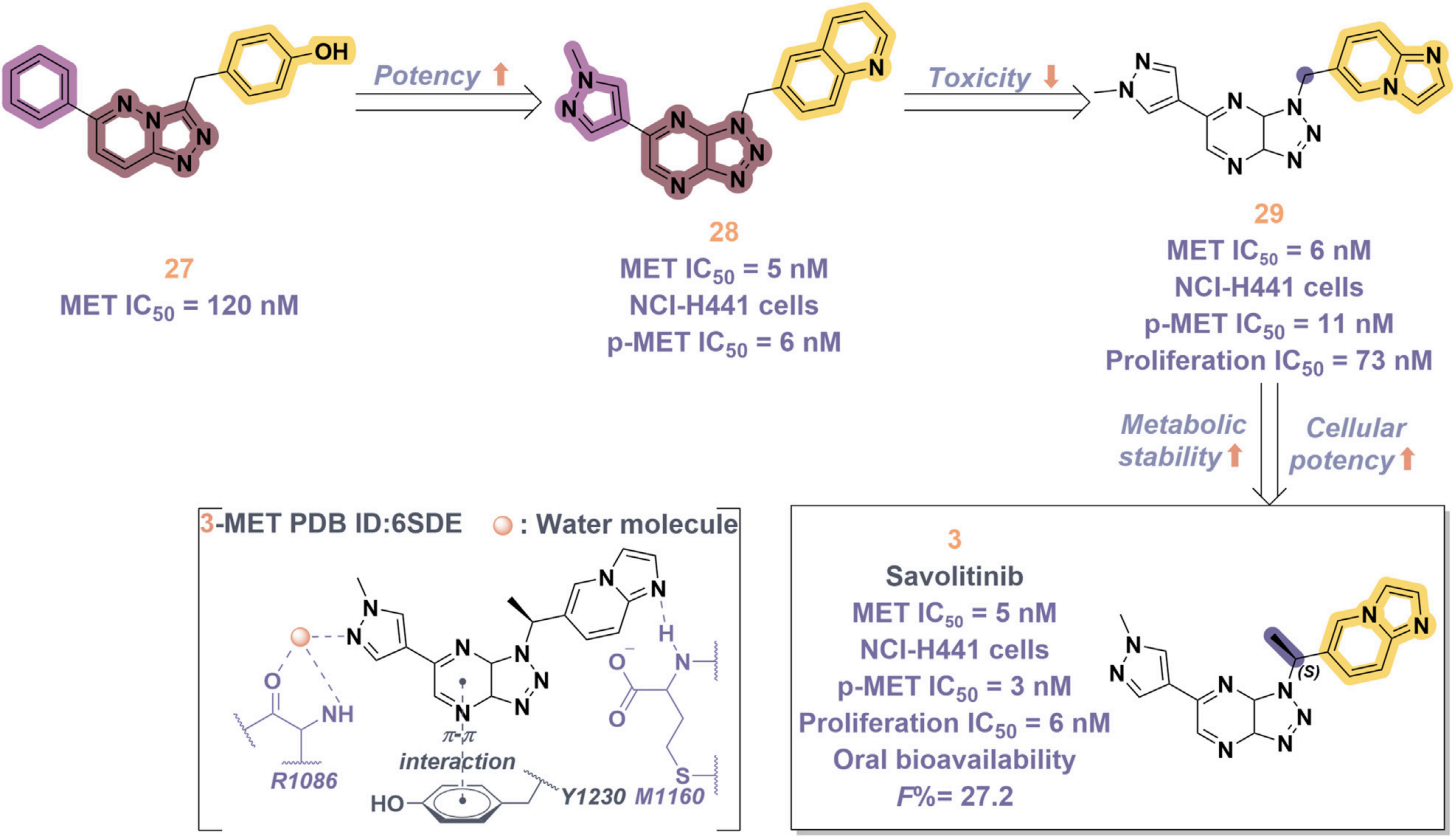
The identification and SAR studies of savolitinib (**3**) are presented. The essential amino acid residues that mediate interactions between the small molecule and the MET protein are depicted. Mauve dashed lines represent hydrogen bonds with key amino acids, and faint gray dashed lines indicate π-π interactions.

Savolitinib (**3**) is a strong MET kinase inhibitor belonging to the triazolopyrazine class. In 2021, marketing authorization has been approved in China for the therapeutic use in adult patients diagnosed with locally advanced or metastatic NSCLC exhibiting MET exon 14 skipping mutations (Pal et al., 2021). This authorization specifically pertains to individuals who have experienced disease progression following platinum-based chemotherapy or who are intolerant to standard platinum-based treatment regimens (Lu et al., 2021). At the molecular level, **3** demonstrates significant inhibitory activity against the MET kinase, exhibits exceptional kinase selectivity, and possesses a strong affinity for the kinase. In the realm of drug discovery, **3** was derived from compound **27** following multiple iterations of structural optimization (Figure 6). Notably, the phenolic group in compound **27** was substituted with a quinoline moiety, while the triazolopyridine group was replaced with a triazolopyrazine moiety, leading to the identification of **28**. In comparison to **27**, the enzyme inhibitory activity of **28** was significantly improved. Consequently, imidazopyridine derivatives were employed to optimize the quinoline moiety in **28**, aiming to mitigate the potential toxicity and side effects associated with the quinoline group, which led to the identification of **29** (Baschnagel et al., 2021). The subsequent structural optimization process aimed to enhance cellular activity and pharmacokinetic properties by incorporating an (*S*)-methyl group at the methylene linkage of **29**, ultimately yielding **3**. The co-crystallization of **3** with MET (PDB code:6SDE) reveals that compound 3 adopts a U-shaped conformation within the binding cavity. The [1,2,3]triazolo [4,5-*b*]pyrazine core facilitates substantial π-stacking interactions with the critical residue Y1230. Additionally, the methyl-substituted pyrazole moiety engages in hydrogen bonding interactions with R1086, mediated by water molecules, while the imidazo [1,2-*a*]pyridine group forms hydrogen bonds with M1160. Additionally, the (*S*)-methyl group of **3** extends into the hydrophobic cavity created by the residues L1157, K1110, and V1092, thereby enhancing the molecule’s binding affinity to MET. *In vivo* pharmacokinetic studies demonstrated that **3** exhibited a satisfactory oral bioavailability (*F*% = 27.2) (Yoh et al., 2021).

**FIGURE 7:**
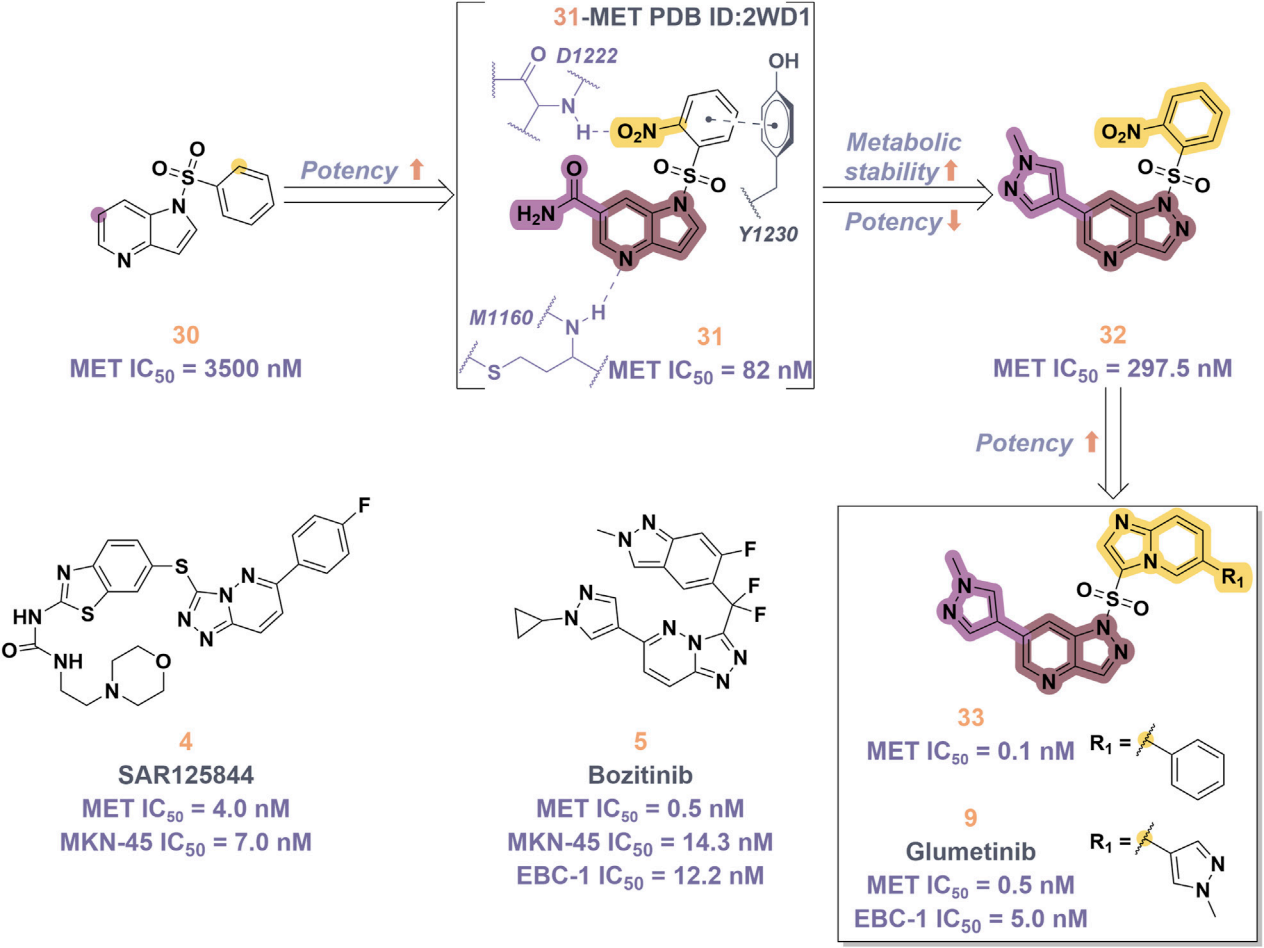
The identification and SAR studies of glumetinib (**9**) are presented. The essential amino acid residues that mediate interactions between the small molecule and the MET protein are depicted. Mauve dashed lines represent hydrogen bonds with key amino acids, and faint gray dashed lines indicate π-π interactions. The chemical structures of candidate molecules SAR125844 (**4**) and bozitinib (**5**), currently undergoing clinical phase I/II studies, are presented.

In 2023, glumetinib (**9**) received approval from the National Medical Products Administration (NMPA) for commercial distribution as a therapeutic agent for adult patients diagnosed with locally advanced or metastatic NSCLC characterized by MET exon 14 skipping mutations (Halder et al., 2024). Structurally, **9** functions as a MET inhibitor, incorporating a pyridine-pyrazole moiety that serves as the ligand for the hinge region of the MET kinase. This compound was developed from **30** through a series of iterative structural optimization processes (Figure 7) (Ai et al., 2018). Initially, **30** underwent structure-activity relationship studies, resulting in the development of **31**, which demonstrated enhanced inhibitory efficacy against the MET kinase. The co-crystallization analysis of **31** with MET (PDB code: 2WD1) demonstrates that the presence of the sulfonyl group within the structure of **31** facilitates the interaction between the two aromatic ring systems, resulting in a U-shaped conformation. Consequently, due to the susceptibility of the pyridine-pyrrole moiety in the structure of **31** to hydroxylation at the nitrogen position *in vivo*, it has been substituted with a pyridine-pyrazole moiety to yield **32**. To enhance the π-π interaction between the small-molecule ligand and the Y1230 residue of MET, the nitrobenzene moiety in **32** was substituted with a 6-phenyl-imidazo [3,2-*a*]pyridine group. This modification led to the identification of MET inhibitor **33**, which demonstrated picomolar inhibitory activity. Subsequently, to enhance the druggability of the compound, the phenyl group in the structure of **33** was substituted with a 1-methyl-1*H*-pyrazole group, ultimately yielding **9**. Simultaneously, other nitrogen-containing heterocycles, including thiazole and indazole, may also be utilized in the design of type Ib inhibitors, exemplified by SAR125844 (**4**) (Egile et al., 2015) and bozitinib (**5**) (Hu et al., 2023). As illustrated in Table 1, compounds **4** and **5** are presently undergoing clinical phase I/II trials and exhibit promising prospects for further development.

**FIGURE 8:**
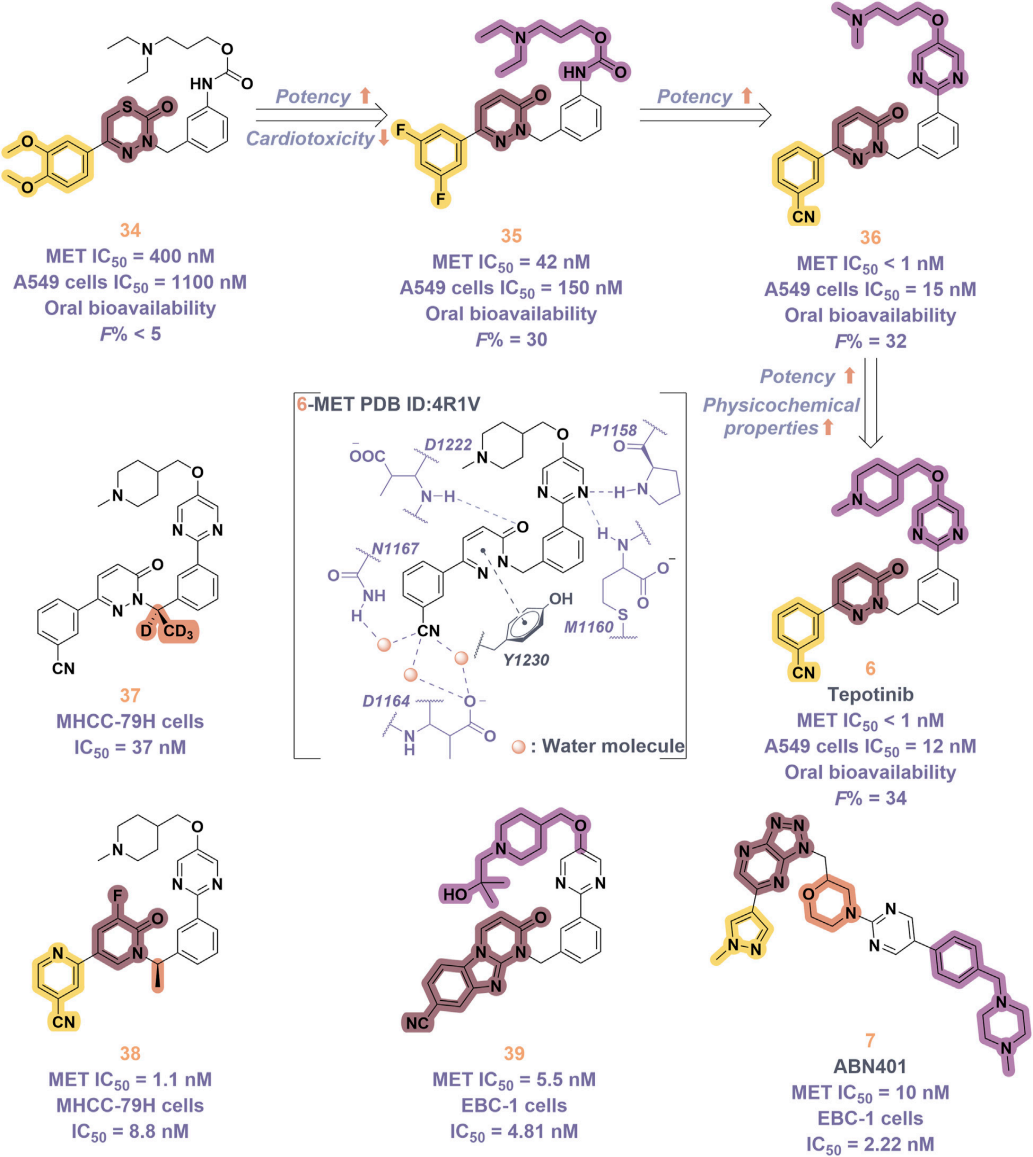
The identification and SAR studies of tepotinib (**6**) are presented. The essential amino acid residues that mediate interactions between the small molecule and the MET protein are depicted. Mauve dashed lines represent hydrogen bonds with key amino acids, and faint gray dashed lines indicate π-π interactions. The chemical structures of tepotinib-based derivatives **37–39**, as well as ABN401 (**7**), are depicted.

Tepotinib (**6**), a highly selective oral MET inhibitor, is designed to target the oncogenic signaling of the MET receptor associated with variations in the MET gene (Albers et al., 2023; Wu Y. L. et al., 2021). In 2023, the NMPA of China granted marketing authorization for **6** for the treatment of NSCLC patients with MET exon 14 skipping mutations (Mazieres et al., 2023). Structurally, **6** is categorized as a small molecule inhibitor, distinguished by the presence of a single pyrimidine group that serves as the ligand for the hinge region of the MET. This compound was developed through several iterations of structural optimization derived from molecule **34** (Figure 8). In compound **34**, the dimethoxy-substituted phenyl group and the thiadiazolone nucleus were replaced with a difluoro-substituted phenyl group and a pyridinone nucleus, respectively, resulting in the formation of compound **35**. This modification resulted in enhanced therapeutic efficacy and reduced cardiotoxicity. However, amino acid methyl esters demonstrate potential metabolic toxicity *in vivo*; thus, further optimization of these compounds is warranted (Wu Z. et al., 2021). Consequently, building upon the original structure, a comprehensive investigation into the structure-activity relationship was undertaken. Compounds **6** and **36** were identified by substituting the amino acid methyl ester with a pyrimidine moiety that is modified with hydrophilic groups, and by replacing the difluoro-substituted phenyl group with a 3-cyanophenyl group. Research findings demonstrate that **6** exhibits low nanomolar enzymatic and cellular inhibitory activities, in addition to possessing favorable oral bioavailability (Jing et al., 2022). To date, the derivatization of **6** has primarily been documented with the aim of enhancing the *in vivo* metabolic stability of the drug, including derivatives **37**, **38**, **39**, and ABN401 (**7**) (Kim et al., 2020). Notably, these derivatives demonstrate improved *in vivo* stability and pharmacokinetic profiles relative to **6**. Among these derivatives, **7** is distinguished by its notable selectivity and advantageous pharmacokinetic properties. It is currently in Phase I/II clinical trials for the treatment of several solid tumors, including NSCLC (Wu Y. et al., 2020).

Macrocycles, defined as cyclic small molecules or peptides featuring ring structures that consist of 12 or more atoms, have gained recognition as promising chemical scaffolds in the domain of drug discovery (Garcia Jimenez et al., 2023). Their unique physicochemical properties, which include a high molecular weight and a significant number of hydrogen bond donors, distinguish them from linear analogues. In contrast to linear analogues, macrocyclic compounds exhibit a tendency to adopt pre-organized and constrained conformations, facilitating extensive interactions with target proteins (Cummings and Sekharan, 2019). As a result, these compounds are likely to exhibit enhanced binding affinity, improved selectivity, and superior pharmacological properties. Recently, the domain of macrocyclic pharmaceuticals has attracted considerable interest from medicinal chemists. Notably, over the past decade, there has been a substantial increase in the number of macrocyclic drugs approved by the FDA that were developed utilizing rational drug design methodologies (Acharya et al., 2023).

**FIGURE 9:**
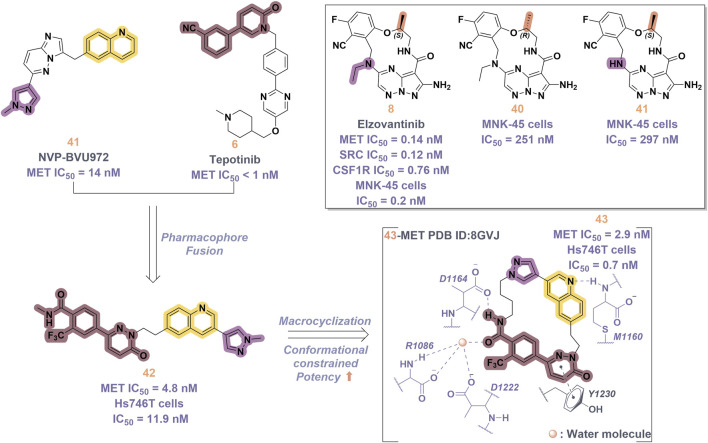
The identification and SAR studies of compound **43** are presented. The essential amino acid residues that mediate interactions between the small molecule and the MET protein are depicted. Mauve dashed lines represent hydrogen bonds with key amino acids, and faint gray dashed lines indicate π-π interactions. The chemical structures of elzovantinib (**8**), along with its derivatives **40** and **41**, are illustrated.

In 2019, a novel macrocyclic inhibitor targeting MET, SRC, and CSF1R, designated as elzovantinib (**8**), was reported, demonstrating picomolar-level inhibitory activity against these kinases. Currently, **8** is undergoing Phase I/II clinical trials in patients with NSCLC who have not previously received treatment with tyrosine kinase inhibitors (Qu et al., 2023). Preliminary results indicate that patients exhibit good tolerance to the treatment and show promising anti-tumor activity. It is noteworthy that the chirality of the methyl groups within the linker region significantly affects the cellular activity of **8**. In comparison, the cellular activities of its enantiomer **40** and the deethylated derivative **41** are diminished by approximately 1,000-fold (Figure 9). This disparity may be ascribed to the influence of the spatial arrangement of atoms on the recognition of drug molecules by protein receptors. In particular, the (*S*)-methyl group within the structure of compound **8** appears to play a crucial role in constraining its conformation (Bouattour et al., 2018). In 2022, Lu and colleagues developed and synthesized a series of “U”-shaped MET inhibitors employing a pharmacophore hybridization strategy, guided by the chemical structures of NVP-BVU972 (**41**) and compound **6** (Figure 9) (Wang C. et al., 2022). Notably, **42** demonstrated significant inhibitory activity against the MET kinase and exhibited pronounced anti-proliferative effects on NSCLC cells. Subsequently, the research team implemented a macrocyclization strategy, which led to the discovery of several macrocyclic inhibitors of the MET kinase. Notably, **43** demonstrated the most promising enzymatic and cellular inhibitory activities, exhibiting an IC_50_ of 2.9 nM for the MET kinase and 0.7 nM for Hs746T cells. However, the pharmacokinetic properties of this compound are suboptimal, indicating the potential for structural refinement. Specifically, in the design of linker groups for macrocyclic inhibitors, it is imperative to consider not only the feasibility of synthesis but also the significant role these linker groups play in facilitating the interaction between the ligand and its target (Amrhein et al., 2021).

### 4.3 Type II inhibitors of MET

Structurally, the occupation of less conserved hydrophobic pockets surrounding the ATP-binding site by type II inhibitors facilitates the binding of small molecules to the protein, thereby enhancing selectivity (Zhao S. et al., 2021). Table 1 provides a summary of the representative type II MET inhibitors that have been incorporated into the clinical treatment of NSCLC.

**FIGURE 10:**
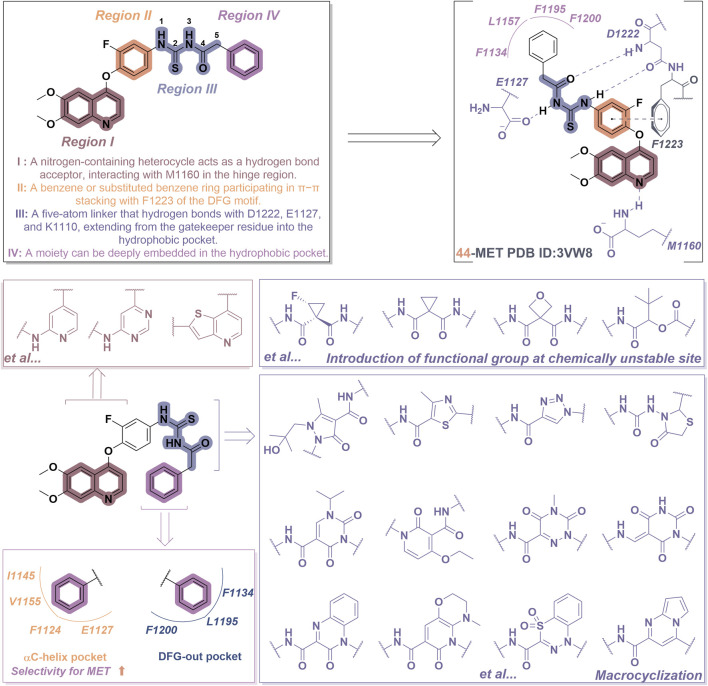
The chemical structure and binding mode characteristics of type II MET inhibitors in their interaction with the Met kinase were elucidated using compound **44** as a representative example. Mauve dashed lines represent hydrogen bonds with key amino acids, and faint gray dashed lines indicate π-π interactions. The strategy for the structural optimization of type II MET inhibitors has been elucidated.

Consider compound **44** as a representative example. As illustrated in Figure 10, most type II inhibitors of MET exhibit certain defining characteristics: (i) Various nitrogen-containing aromatic heterocycles (region I), including pyridine, pyrrolidine, and quinazoline, can be incorporated to establish hydrogen bonds with the amino acid residue Met1160 located in the hinge region of MET. Furthermore, the side chains of these aromatic heterocycles play a crucial role in modulating the affinity of the molecule for its target; (ii) Unsubstituted or mono-substituted pyridine and benzene rings (region II) possess the capacity to participate in π-π interactions with the residue F1223; (iii) The incorporation of a flexible chain or rigid ring structure, specifically a five-atom linking group, that includes one or more hydrogen bond donors or acceptors (designated as region III), significantly enhances the molecular potency. This region promotes the establishment of a hydrogen bond network with the amino acid residues of the MET kinase, including Asp1220, Lys1110, and Leu1245, among others; (iv) The region IV segment consists of six-membered aromatic heterocycles that may be unsubstituted, mono-substituted, or di-substituted, and this segment extends into the hydrophobic pocket of the MET kinase (Redmer et al., 2024).

The recently reported novel type II inhibitors of MET primarily focus on the optimization of regions I and III. In the context of region III, it is significant to note that one optimization strategy entails the identification of novel linkers that comply with the “5-atom rule” (Nan et al., 2020). This objective can be accomplished by introducing cyclopropyl and oxy groups at chemically unstable sites or by replacing these groups with alternative linear structures, such as ester groups, sulfone groups, and double bonds, as illustrated by compounds **10**, **11**, and **12** in Table 1 (Nan et al., 2023). Furthermore, an alternative optimization strategy entails the cyclization approach; however, it is crucial to verify that the region IV component can adequately extend into the hydrophobic pocket, as evidenced by compounds such as **13**, **14**, and **15**. Macrocyclization can yield five-membered, six-membered, and bicyclic heterocycles. The macrocyclic structures described above have the ability to form hydrogen bond networks with the amino acid residues present in the kinase domain of the target protein (Bauder et al., 2021). Furthermore, region IV is highly likely to extend into the DFG-out conformation pocket, which comprises residues F1200, L1195, and F1134. Notably, the region IV of certain inhibitors may extend into the αC-helical pocket formed by the residues I1145, V1155, F1124, and E1127 (Figure 10). This pocket is infrequently observed in kinases other than the MET kinase, which consequently allows these inhibitors to demonstrate a high degree of selectivity for the MET kinase (Pievsky and Pyrsopoulos, 2016). In light of the significant advancements in research on type II inhibitors, several comprehensive reviews are currently available (Liu et al., 2022; Jin et al., 2024); consequently, a detailed discussion on this topic will not be undertaken in this context.

### 4.4 Type III inhibitors of MET

**FIGURE 11:**
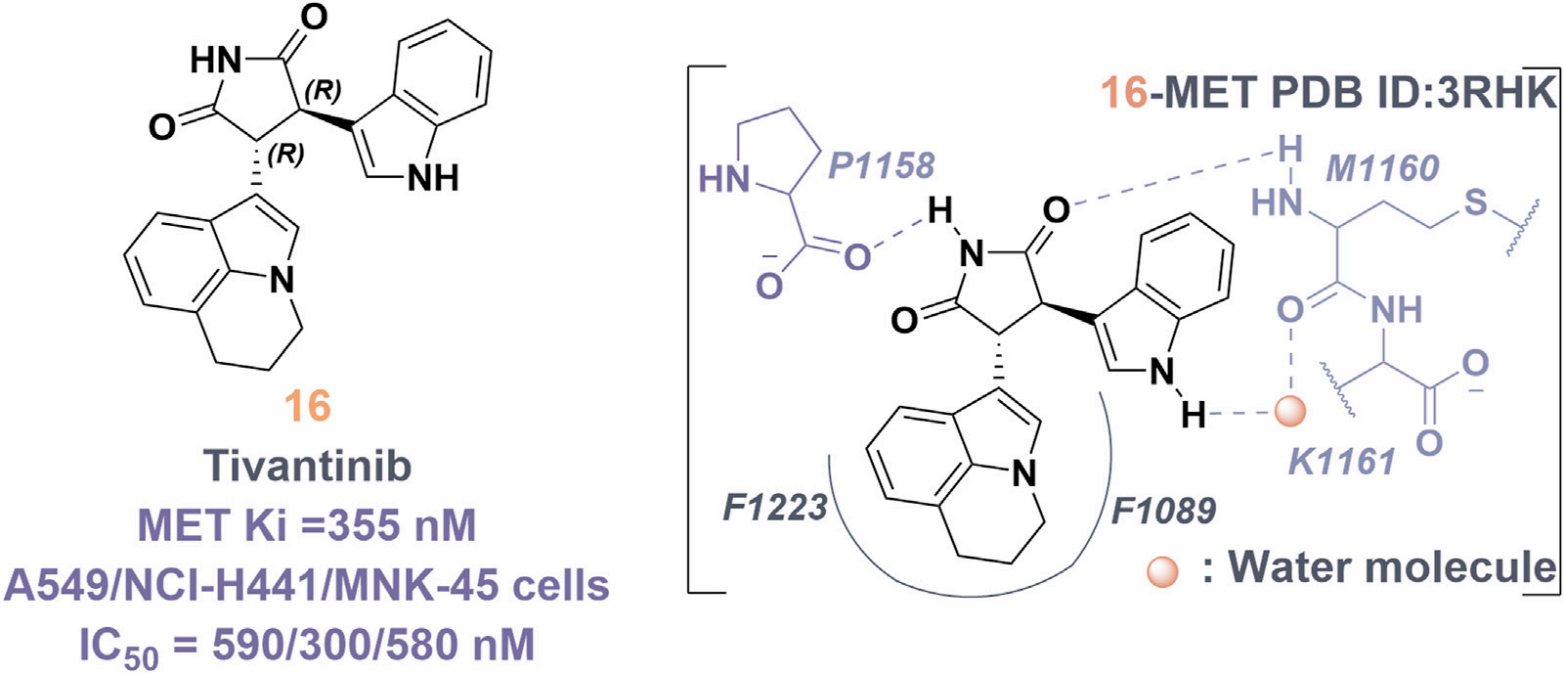
The chemical structure and binding mode characteristics of tivantinib (**16**) in their interaction with the unphosphorylated MET kinase domain were elucidated. Hydrogen bonds are represented by mauve dashed lines connecting to the key amino acids.

Tivantinib (**16**) is classified as a type III inhibitor of Met. *In vitro* studies have demonstrated that **16** possesses an inhibitory constant (Ki) of approximately 355 nM, indicating a high degree of selectivity for the MET kinase when compared to 230 other human-derived kinases evaluated concurrently (Eathiraj et al., 2011). The co-crystal structure of compound **16** in complex with the unphosphorylated MET kinase (PDB ID: 3RHK) elucidates the formation of hydrogen bonds between the pyrrolidinylindolone fragment and the amino acid residues P1158 and M1160 (Figure 11). Additionally, the tricyclic moiety demonstrates a perpendicular orientation in relation to the indole ring within the binding site, subsequently extending into the hydrophobic cavity situated between the F1223 residues and the F1089 residues in MET kinase. The binding mechanism of **16** to the unphosphorylated MET receptor specifically stabilizes the “DFG-out” conformation, concurrently disrupting the salt bridge between residues E1127 and K1110 (Calles et al., 2015). Protein kinases demonstrate a significant degree of conservation in their three-dimensional structures, especially in the regions surrounding the ATP-binding site. Presently, most kinase inhibitors target this ATP-binding site, which often leads to challenges related to selectivity and off-target effects. Compound **16** is notable for its ability to occupy the hydrophobic pocket created by the displacement of the α-C helix in MET kinase. This modification alters the binding site’s conformation, transforming the polar ATP-binding pocket into a non-polar environment, making it unsuitable for ATP (Yoshioka et al., 2015).

Preliminary clinical research suggests that the combination of **16** and erlotinib may significantly extend the survival duration of patients with refractory NSCLC. A Phase III clinical trial (NCT01377376) investigated the therapeutic efficacy of erlotinib in conjunction with tivantinib in an Asian cohort of patients diagnosed with stage IIIB/IV EGFR wild-type, non-squamous NSCLC (Han et al., 2024). The trial was prematurely terminated due to an increased incidence of interstitial lung disease (ILD) observed in the group treated with **16**. Before its discontinuation, the clinical data demonstrated no statistically significant difference in median overall survival (OS) between the combination therapy group and the erlotinib monotherapy group. Despite the challenges encountered in the clinical development of **16**, its unique mechanism of action has provided valuable insights for the subsequent identification of novel targeted therapeutic strategies aimed at the MET kinase in NSCLC.

## 5 Resistance mechanism of MET inhibitors

In recent years, several small molecule inhibitors that target the MET gene have been introduced to the market, offering survival advantages for numerous patients with NSCLC who possess MET gene mutations (Huang et al., 2019). However, although certain patients may initially exhibit therapeutic benefits, as indicated by tumor stabilization or reduction, these advantages are frequently temporary, resulting in subsequent tumor relapse and disease progression. Additionally, a subset of patients does not experience any therapeutic benefit from MET inhibitors. Specifically, drug resistance can be broadly classified into primary resistance and acquired resistance (Sun et al., 2022).

### 5.1 Primary resistance

**FIGURE 12:**
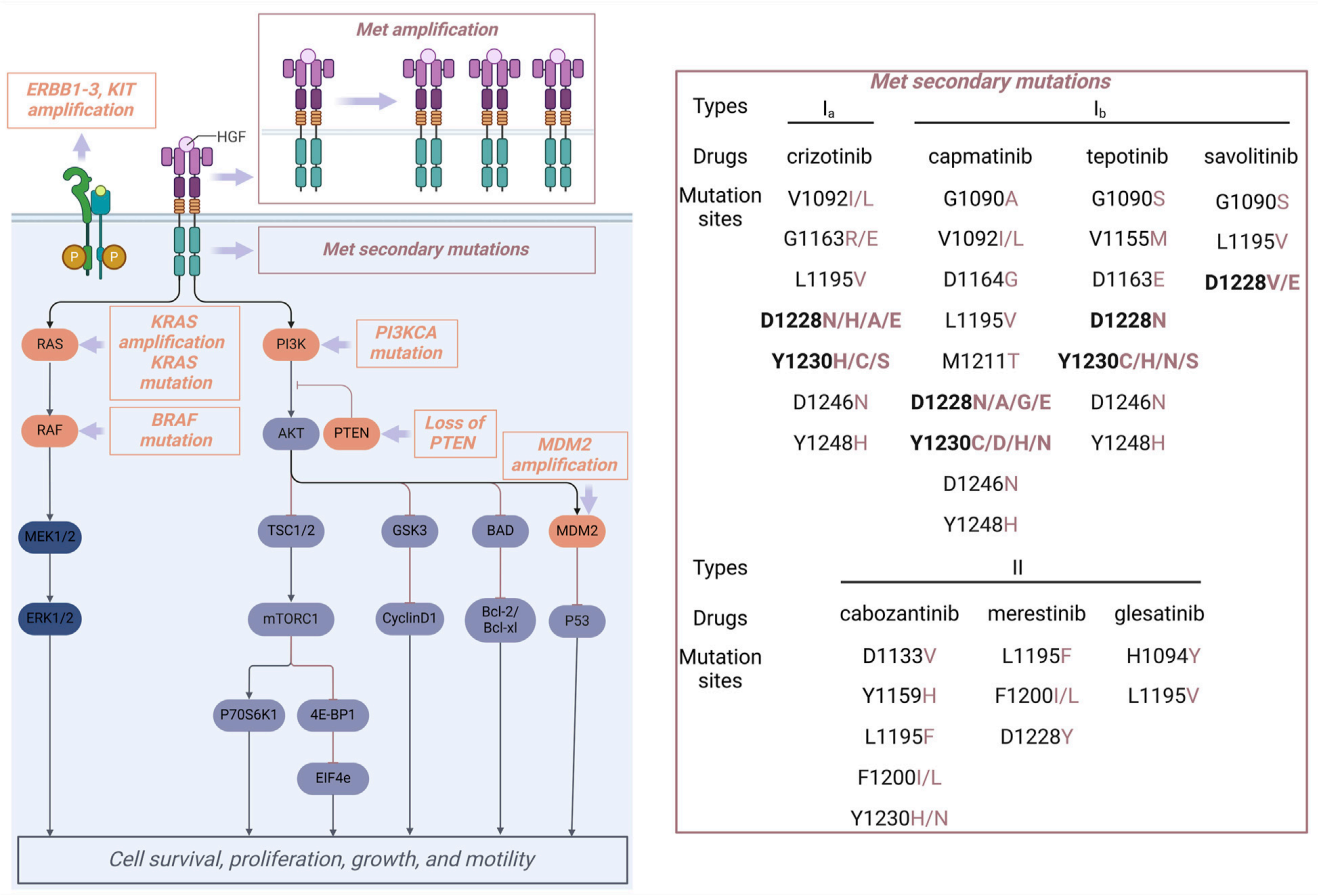
Reported primary drug resistance (orange) and acquired drug resistance (brownish-red) mechanisms for MET inhibitors.

Primary drug resistance is characterized by the inability of patients with MET alterations to exhibit an initial therapeutic response to MET inhibitors. Mechanistic investigations indicate that primary drug resistance is primarily attributable to the presence of activating mutations in bypass or downstream signaling pathways within tumor cells, particularly involving the activation of the RAS/MAPK pathway and alterations in the PI3K-AKT pathway (Figure 12) (Zhang Y. et al., 2021). Research has demonstrated that the co-occurrence of EGFR amplification, KRAS mutation, BRAF mutation, TP53 mutation, MDM2 amplification, PTEN protein loss, and PIK3CA mutation or amplification is prevalent in tumors exhibiting MET exon 14 skipping mutations. This coexistence of mutations is associated with primary resistance to MET inhibitors and a reduced duration of response to these therapies in affected patients (Zhou X. et al., 2021; Zhou J. et al., 2021). Preclinical investigations have demonstrated that the concurrent administration of MET inhibitors and PI3K inhibitors can effectively reestablish the sensitivity of drug-resistant tumor cells to MET inhibitors. Therefore, the concomitant use of MET inhibitor and PI3K inhibitors represents a viable approach to overcoming primary resistance to MET inhibitors (Fujino et al., 2019).

### 5.2 Secondary resistance

Following the successful initial treatment with a MET inhibitor, novel structural alterations in the MET domain may arise, leading to the development of secondary resistance. Secondary resistance is typically classified by its mechanisms into MET-dependent resistance, which accounts for about one-third of cases and includes secondary mutations and amplifications in the MET gene as well as HGF gene amplification, and non-MET-dependent resistance, characterized by aberrant activation of downstream signaling pathways related to MET and alternative bypass pathways (Recondo et al., 2020). Within the framework of MET-dependent secondary resistance, mutations at secondary sites within the MET kinase domain represent the most frequently observed alterations. Figure 12 provides a summary of the secondary MET mutation sites that have been documented in response to treatment with MET inhibitors. Specifically, the Y1230 and D1228 residues are key mutation sites for type I inhibitors, whereas L1195 and F1200 residues are mainly associated with type II inhibitors (Marona et al., 2019). Research in structural biology has elucidated that the interaction between Y1230 and type I molecules is pivotal for their efficacy, with mutations at the Y1230 position leading to a marked reduction in potency. Additionally, the conformation of residue Y1230 is closely linked to the salt bridge between D1228 and K1110. Consequently, the mutation at the D1228 site is highly likely to disrupt the integrity of the salt bridge, consequently altering the conformation of Y1230 and diminishing the binding affinity of the inhibitor to the target protein. Type II inhibitors interact with MET in a non-active “DFG-out” conformation, which does not depend on the π-π interaction with Y1230 (Sweeney et al., 2023). Consequently, these inhibitors retain their efficacy in patients with mutations at D1228 and Y1230. Residues L1195 and F1200 are crucial for type II MET inhibitors’ effectiveness due to their key positions in the hydrophobic pocket of region IV (Figure 10). In the “DFG-out” conformation, mutations at residues L1195 and F1200 will affect the sensitivity to type I inhibitors. Additionally, mutations at residue G1163, located within the solvent-exposed region, will hinder the binding of type Ia inhibitors within the hydrophobic pocket, thereby promoting drug resistance; however, these mutations will not influence the efficacy of type Ib and type II inhibitors of the MET kinase (Li et al., 2017). Clinical studies have demonstrated that secondary mutations arising from the administration of type I MET inhibitors can lead to acquired resistance, which may be effectively managed through the use of type II MET kinase inhibitors or by employing reverse strategies. This conclusion has been substantiated by clinical case analyses (Parsons et al., 2020). However, there exists a probability that the transition from type I to type II inhibitors consistently yields unsatisfactory outcomes for mutant variants. Especially, some patients harboring the MET^D1228N^ resistance mutation demonstrate insensitivity to type II inhibitors, including **10** (Xu et al., 2022). Consequently, following the emergence of resistance to MET inhibitors, elucidating the underlying resistance mechanisms through secondary biopsy and second-generation sequencing techniques is essential for informing the subsequent treatment strategy.

The resistance mechanism that operates independently of MET is predominantly mediated by aberrantly activated bypass signaling pathways linked to the ErbB family of proteins (Lei et al., 2023). Additionally, alterations in the RAS/RAF/MEK pathway and modifications in the PI3K/AKT/mTOR pathway contribute to this phenomenon (Figure 12). Combination therapy strategies have been shown to effectively counteract non-MET-dependent resistance mechanisms (Lombardi et al., 2024). Especially, tumors harboring KRAS gene mutations or amplifications may be effectively managed through a combination therapy approach that incorporates MET inhibitors. This strategy includes the concurrent administration of MET inhibitors with PI3K inhibitors, MEK inhibitors, or EGFR inhibitors.

## 6 Perspectives and conclusion

The MET gene assumes a vital role in the progression of NSCLC. MET inhibitors have manifested significant effectiveness in patients harboring MET gene mutations (Yang et al., 2021). Nevertheless, despite the considerable advancements achieved in the development of MET inhibitors to date, the future progression of these therapeutic agents continues to encounter numerous challenges.

Firstly, the prevalent adverse reactions associated with MET-targeted therapies include peripheral edema, nausea and vomiting, and hepatotoxicity. Mechanistic investigations suggest that MET inhibitors disrupt the HGF/MET signaling pathway, which may compromise the permeability balance of vascular endothelial cells, consequently leading to tissue edema (Ayoub et al., 2021). Furthermore, the activity of metabolic enzymes *in vivo* may facilitate the conversion of MET inhibitors into active metabolites, which can subsequently interact with biological macromolecules within the organism. This interaction may lead to structural alterations and functional modifications of these macromolecules, potentially resulting in toxic effects and adverse reactions. Therefore, in clinical practice, the mechanisms underlying the toxicity induced by Met inhibitors are adjusted to minimize organ damage.

Secondly, the persistent challenge of drug resistance necessitates ongoing scrutiny. Especially, Mutations at residues Y1230 and D1228 disrupt the π−π interactions between the small-molecule ligand and the MET kinase, consequently abrogating the inhibitory effects of the ligand. To address drug resistance caused by kinase domain mutations, the common clinical practice is to alternate between type I and type II inhibitors, though this method is not always successful. For example, certain patients exhibiting the D1228N mutation demonstrate insensitivity to type II inhibitors, including cabozantinib (**10**). Consequently, there exists an urgent necessity to develop novel MET inhibitors to address the issue of targeted drug resistance in clinical practice (Lu et al., 2020). The following outlines feasible strategies for overcoming resistance to MET-targeted therapy:

(i) Research indicates that rational drug design focused on targeting mutant sites constitutes a pivotal strategy for identifying inhibitors that can effectively overcome MET resistance (Ferng et al., 2022). For example, the substitution of large-volume hydrophilic groups, such as 4-piperidylmorpholine, with small hydrophilic groups, including five-membered nitrogen heterocycles like pyrazole, isoxazole, furan, and thiophene, mitigates steric hindrance associated with solvent-exposed site mutations and may facilitate the formation of additional interactions. Moreover, the conformational constraints imposed by macrocyclization can mitigate entropy loss during ligand binding, enhance inhibitory activity, and simultaneously minimize steric hindrance in the solvent front region.
(ii) The D1228N mutation is commonly identified in patients undergoing treatment with type I MET inhibitors, such as crizotinib, capmatinib, and tepotinib; however, these patients exhibit insensitivity to type II MET inhibitors. Mechanistic studies suggest that the mutation at this site alters the electrostatic potential of the solvent-exposed region. In light of this alteration, and in accordance with the design strategy employed for the FLT3 inhibitor FF-10101, a small molecule inhibitor specifically targeting MET^D1228N^ was identified through a covalent drug design approach (Ma et al., 2024). Among these inhibitors, electrophilic groups (including acrylamide, epoxides, and halocarbonyl group) are present, which can form covalent bonds with nucleophilic residues such as cysteine or lysine at the active site (Huang et al., 2023). Unfortunately, no covalent inhibitors targeting MET have been reported to date.
(iii) The design of inhibitors targeting allosteric sites, or pockets, located in non-active regions of proteins is undertaken. In contrast to ATP-binding sites, these allosteric pockets exhibit a lower degree of conservation, suggesting that inhibitors directed at them may possess enhanced selectivity (Yan et al., 2016). For example, tivantinib (**16**) occupies the hydrophobic pocket created by the translocation of the α-C helix of Met, thereby functioning as an allosteric binding site.
(iv) Targeted protein degradation (TPD) represents an innovative approach to protein degradation that has garnered significant attention within the realms of chemical biology and drug discovery. Unlike the “occupation-driven” mechanisms characteristic of conventional small molecule inhibitors, TPD operates through an “event-driven” pharmacological mechanism, facilitating the degradation of target proteins via endogenous protein clearance pathways. This distinctive strategy offers several advantages, including its efficacy with minimal catalytic doses, the ability to circumvent established drug resistance mechanisms and off-target effects, the degradation of “undruggable” targets, and the capacity to target the entire protein framework to modulate both catalytic and non-catalytic functions of proteins. Furthermore, it has been successfully utilized in the development of novel anti-tumor therapeutics. Recently, Min et al. developed a series of MET-targeting degraders utilizing hydrophobic tag degradation technology, informed by the structural characteristics of **6** (Min et al., 2024). Their research demonstrated that the candidate molecules effectively inhibited tumor growth both *in vivo* and *in vitro* by facilitating the degradation of MET. In the Ba/F3-TPR-MET^D1228N^ and Ba/F3-TPR-MET^Y1230H^ drug-resistant cell lines, the candidate molecule exhibited enhanced potency relative to **6.** This study further substantiates the efficacy of targeted protein degradation strategies in addressing MET resistance.
(v) In consideration of the complex resistance mechanisms associated with MET inhibitors, which encompass a range of factors and signaling pathways, the strategic selection and implementation of diverse combination therapies may enhance the potential to overcome drug resistance. As shown in Table 1, numerous clinical trials have reported the utilization of MET inhibitors, either alone or in combination with other tumor-targeted therapies, for the treatment of various types of cancer. A substantial body of preclinical and clinical evidence has demonstrated that the combination of MET inhibitors with various tumor-targeted agents, including epigenetic therapies, immunotherapeutics, and other RTK inhibitors, exhibits synergistic effects. However, in the context of combination therapy, it is imperative to take into account several factors, including the cumulative dosage of the medications, the complex pharmacokinetic properties of the two drugs, and the potential for toxicities and adverse effects (Zhou X. et al., 2021). Alternatively, pharmacological agents that target dual or multiple pathways may reduce the risk of drug-related adverse reactions, demonstrate enhanced pharmacokinetic profiles, and improve overall safety.

Collectively, this article provides a thorough review of recent advancements in research concerning MET inhibitors for the treatment of NSCLC. It offers an in-depth analysis of the pathogenesis of MET in NSCLC, as well as the current progress in the development of MET inhibitors. These findings are anticipated to facilitate the research and development of future MET inhibitors that exhibit high efficacy, selectivity, and low toxicity.

## Abbreviations

ALK, anaplastic lymphoma kinase; BRAF, oncogene homolog B1; EGFR, epidermal growth factor receptor; HER2, human epidermal growth factor receptor 2; HGF, hepatocyte growth factor; IPT domain, immunoglobulin-plexin-transcription factor domain; IRAC, the International Agency for Research on Cancer; MET, mesenchymal-epithelial transition factor; NCCN, The National Comprehensive Cancer Network; NSCLC, non-small cell lung cancer; PSI domain, the plexin-semaphorin-integrin domain; ROS1, c-ros oncogene 1 tyrosine kinase; RTK, receptor tyrosine kinase; SCLC, small cell lung cancer; TPD, targeted protein degradation.
